# *ReSVA*: A MATLAB method to co-register and mosaic airborne video-based remotely sensed data

**DOI:** 10.1016/j.mex.2021.101471

**Published:** 2021-07-27

**Authors:** Tomas Naprstek, J. Pablo Arroyo-Mora, Joshua M. Johnston, George Leblanc

**Affiliations:** a Flight Research Laboratory, Aerospace Research Centre, National Research Council Canada, ON, Canada; b Canadian Forest Service, Natural Resources Canada, ON, Canada

**Keywords:** Geocorrection, Gridding, Airborne survey, Image processing

## Abstract

Airborne remotely sensed data (*e.g*. hyperspectral imagery, thermal videography, full frame RGB photography) often requires post-processing to be combined into a series of images or a mosaic for analysis. This is generally accomplished through the use of position and attitude hardware (*i.e*. Global Navigation Satellite System - GNSS / Inertial Measurement Unit - IMU) in combination with specialized software. Occasionally, hardware failure in the GNSS/IMU instrumentation occurs, however the data are still recoverable through a correction process, which allows image registration to mosaic the data. Here we present a simple and flexible MATLAB® code package that has been developed to combine video-based remotely sensed data. It first applies an iterative image registration process to align all frames, using pre-existing GPS information if supplied by the user, and then grids the frame data together to develop a final, single mosaic dataset that can be used for analysis. An example of this method using airborne infrared video data of a wildfire is shown as a demonstration.

• MATLAB functions are easily adaptable to specific user needs and datasets.
• The method outputs the combined data and positional information in three separate MATLAB variables that can be readily used for analysis in MATLAB or exported for use in other software.

## Graphical abstract

**Figure:**
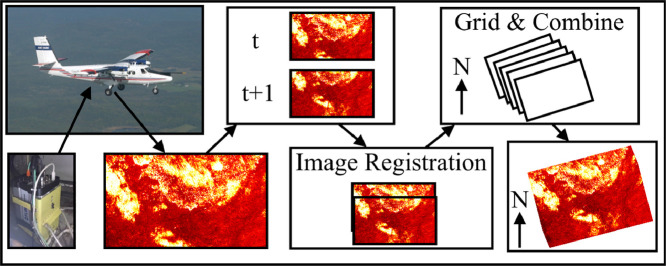

Specification table

**Table:** 

Subject Area	*Earth and Planetary Sciences*
More specific subject area	*Developing combined remote sensing images from video data without attitude information*
Method name	*Remote Sensing Video Alignment (ReSVA) in MATLAB®*
Name and reference of original method	Image registration in the context of airborne video imagery data, such as: J. Le Moigne, N. S. Netanyahu, R. D. Eastman, Image registration for remote sensing, 1^st^ edition, 2011, Cambridge University Press. R. A. Schowengerdt, Remote sensing: models and methods for image processing, 3^rd^ edition, 2007, Elsevier.
Resource availability	*MATLAB (requires the Image Processing Toolbox)*

## Method details

When collecting airborne remotely sensed data (e.g. video, RGB stills, hyperspectral), combining multiple frames of data into a single, usually geocorrected image or mosaic is necessary for analysis [1], [2], [3]. This process requires high accuracy (*e.g*. subject to each application) from an onboard Global Navigation Satellite System (GNSS) and an Inertial Measurement Unit (IMU) [4]. In situations where sensor [5] or human error do not allow for collecting such data, then the resulting imagery's positional information will contain errors of varying amplitudes. However, image registration might still be a valid approach to produce co-registered images, or an image mosaic, under the assumption that the output image or mosaic will provide useful information [*e.g*. 6].

Here we detail a two-part MATLAB process for registering and combining remote sensing video data, or a single sensor with frequently collected imagery, as the registration process assumes very little change from frame to frame. Therefore, this method should be applicable to most airborne-based video data, such as electro-optical, infrared, and multi-spectral cameras collected at a frequency where there is extensive overlap in data from frame to frame. However, it should be noted that this method is reliant upon noticeable inhomogeneity in the data. For instance, in the dataset we provide as a case study, the wildfire being analyzed provides intensity-distinctive features (large temperature contrast between specific fire pixels) which assists in constraining the image registration function. Structurally distinctive elements, such as buildings or rivers also provide contrasting edges in the imagery, assisting in constraining the registration process. A key assumption in this method is that one frame of data contains GNSS positional information, allowing every pixel in the image to be assigned a coordinate location. This is done either through a GNSS-tag or using a known feature within the dataset to get estimated positions. If the coordinate location of the total dataset is not important (or not available), the video data can still be processed into a single image by assigning generic positions to the initial frame (i.e. (*x,y*) = (0,0), (0,1), etc.). By using primarily built-in MATLAB functions (including the Image Processing Toolbox), it can be readily used as-is, or modified for a user's specific purposes with very little effort. Both the code and case study data can be found within the online version's Supplementaty Materials section or at the author's GitHub: https://github.com/TomasNaprstek/ReSVA.

We give an example of our methodology through the usage of infrared video data collected using a 3.0 µm to 5.0 µm broadband Forward-looking Infrared (FLIR) SC8300 camera (FLIR Systems Inc.), modified to be carried in the National Research Council of Canada's Twin Otter aircraft. The flight campaign was completed over an active wildfire in Northern Ontario, Canada, which required an accurate mosaic of the frames for analysis.

## Procedure

### Main script

The two parts of the process are built as separate functions, which are called from a main script. The first step is loading in the data.

clear;

load('raw_ir_data.mat'); %The example data contains variables: ir_data, xpos, and ypos.

frame_data = ir_data; %Assign the example ir_data to the generic frame_data variable.

The data must contain three matrix variables: the remotely sensed video data save as individual frames, and the *x* and *y* positions of the first frame of data in UTM meters (Table 1). The frame matrix must contain the following information: the number of rows, the number columns, and the total number of frames. This assumes that the frame data are of consistent size in terms of the number of rows and columns. The other two matrices, the *x* and *y* positions of the first frame, must also be this same size. These two matrices are used as the initial positions for all other frames to be registered against. For example, the positional data for the initial frame will be a series of *x* and *y* coordinate pairs. As such, a pixel *m* would have position (*x1, y1*), and a pixel *n*, that is directly beside pixel *m*, would have position (*x2, y2*). In this situation, the spacing between these two adjacent pixels is *y2*-*y1* and *x2* - *x1*. These positions must be in a meter-based coordinate system such as UTM.

**Table 1:** A description of the required input variables and their matrix size.

Variables	Matrix Size	Description
*frame_data*	(number of frames, *y* size - # rows, *x* size - # columns)	The raw video data split into individual frames, as a 3-dimensional matrix.
*xpos*	(*y* size - # rows, *x* size - # columns)	The *x* positions (in UTM meters) of all pixels in *frame_data*(1,:,:)
*ypos*	(*y* size - # rows, *x* size - # columns)	The *y* positions (in UTM meters) of all pixels in *frame_data*(1,:,:)

If the frame data are from a sensor that contains more than 1 channel, the data must be "flattened" before registration, or only a single channel must be used for the registration. For instance, if using an RGB sensor, uncomment the following section to convert to grayscale before registering. Afterwards, the gridding function should be run separately for each channel.

% xres = length(frame_data(1,1,:,1));

% yres = length(frame_data(1,:,1,1));

% frame_data_grey = zeros(length(frame_data(:,1,1,1)),yres,xres);

% for i = 1:length(frame_data(:,1,1,1))

% tempframe = reshape(frame_data(i,:,:,:),yres,xres,3);

% frame_data_grey(i,:,:) = rgb2gray(tempframe);

% end

As described above in the Method Details section, the assumption here is that the positional information is generated by the user through either a GNSS-tag, or from geographically known points in the data. For instance, if the data contain a GNSS-tag (e.g. from the aircraft's on-board recorder), the user could provide estimates for all frame data at that time-stamp by assuming the position is in the direct center of the frame (assuming a nadir-pointing sensor), and extrapolating all other positions based on the sensor's lens information and aircraft altitude. Another option would be to find a location within the imagery that contains a clear landmark (either known from ground-based GNSS marking or from finding it in other geocorrected imagery), and use that to extrapolate positions of all data in the image. An easy alternative option for the user, if the geographical position is not required, or will be integrated through another method, would be to assign the two positional matrices *x* and *y* information that is simply the row and column numbers. This can be done by uncommenting the below line of code.

% [xpos, ypos] = meshgrid(1:length(frame_data(1,1,:)),1:length(frame_data(1,:,1)));

Each cell in the first frame matrix must have some positional information for the registration process to use for initialization. This information, along with all frame data, is passed to the first function which registers the frame data, giving *x* and *y* positional information for every pixel in every frame. The output of this function is two matrices of the same size as the input *frame_data* matrix, containing the registered *x* and *y* positions for each frame.

%Register the data.

[reg_x,reg_y] = resva_register(frame_data,xpos,ypos);

Once the registration is complete, the user may optionally plot the result of each registered frame. By looping through the frame data and plotting each as a surface, the pre-gridding result can be seen. This process can be computationally intensive if the frames are large, or if there are many frames to plot. If this is the case, plotting every 5^th^, 10^th^, etc. frame will also accomplish this initial inspection. Also, this plot (see code below) is set up for the example dataset, and therefore the *zlabel* and *caxis* functions are dataset specific.

%Uncomment the section below to see the data before gridding.

%WARNING: if the dataset is large, it may take a long time to plot all data!

%%

% res_x = length(frame_data(1,1,:));

% res_y = length(frame_data(1,:,1));

% figure;

% for i = 1:length(frame_data(:,1,1))

% xvals = reshape(reg_x(i,:,:),res_y,res_x);

% yvals = reshape(reg_y(i,:,:),res_y,res_x);

% vals = reshape(frame_data(i,:,:),res_y,res_x);

%

% surf(xvals,yvals,vals,'edgecolor','none');

% hold on;

% end

% grid on;

% axis equal;

% view([-40 85]);

% xlabel('Eastings (m)');

% ylabel('Northings (m)');

% zlabel('Counts');

% title('Registered Dataset');

% caxis([2050 2150]);

%%

**Fig. 1:**
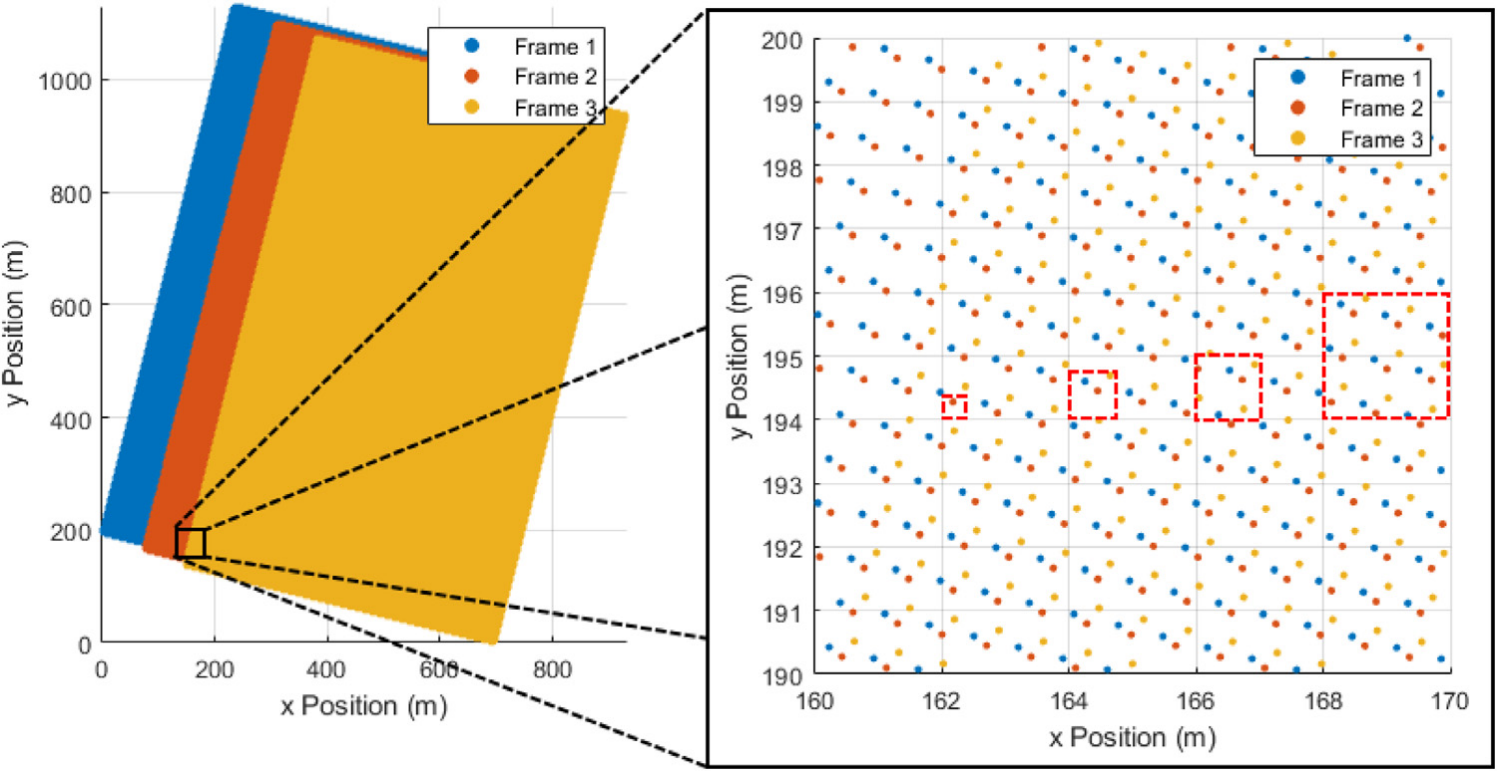
An example of the distribution of pixels across three frames of data that have been registered to each other. The zoomed in portion shows the specific location of the pixels, and which frame they come from. The four dashed red boxes are examples of options for this dataset's grid cell size. From left to right, they are 0.5 m, 0.8 m, 1.0 m, and 2.0 m. The smallest, 0.5 m, is too small, and may lead to “holes” in the final grid. The 0.8 m and 1.0 m sizes are both appropriate, and good choices for the cell size. The 2.0 m size is too large, and will reduce the resolution of the final image.

Before our gridding function is called, two user-defined parameters must be specified: the grid's cell size and the gridding method. The cell size should be the same or larger than the average pixel-to-pixel physical separation to avoid the possibility of “holes” in the data where no pixels fall (Fig. 1). A larger cell size will assist in smoothing over any minor discrepancies that did not align during the registration process, but will result in changes that are more significant to the final grid.

**Fig. 2:**
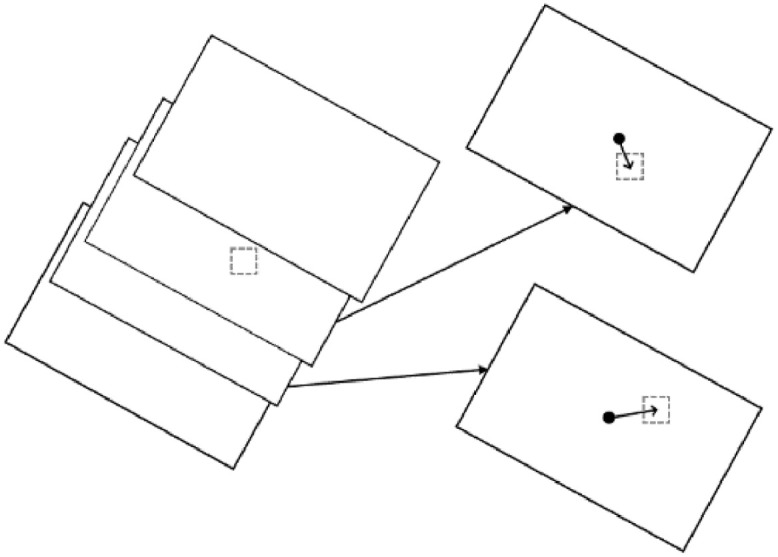
An exaggerated example of the process for choosing which pixel to take data from during the ‘*centerframe*’ gridding method. The large rectangles are the frames of data and the gray dashed box represents the grid cell being analyzed. In this case, two frames contain data that fall within the cell, as shown on the right by the arrows. The upper frame's pixel would be used, as the radial distance from the center of the frame (arrow from center point circle to the cell being analyzed) is smaller than the bottom frame.

The gridding method can be one of two options: either ‘*average*’ or ‘*centerframe*’. The first will simply take the average of every pixel that falls within a given cell. The second option meanwhile will use only a single pixel for each cell, determined by which pixel's original frame's center is closest to the cell (Fig. 2).

%Set up gridding parameters

cell = 1; %subsample to this cell size (in metres)

%Choose a gridding method:

% 'average' = take the average of all data that falls into each cell.

% 'centerframe' = use only the datapoint whose location was closest to

% its origin frame's center (this is useful for cameras/sensors that have

% an accuracy bias towards the center of the frame data).

gridmethod = 'centerframe';

%Grid the data

[grid_data,grid_x,grid_y] = resva_grid(frame_data,reg_x,reg_y,cell,gridmethod);

Once gridded, the main script plots a raster of the completed registered and gridded data. This plotting is set up for the example dataset, and therefore the *zlabel* and *caxis* functions will need to be changed for the user's own specific dataset. At this point the user may export the three variables, *grid_x, grid_y*, and *grid_data*, to be used for further analysis elsewhere.

%Plot the final gridded dataset

figure;

surf(grid_x,grid_y,grid_data,'edgecolor','none');

grid on;

axis equal;

view([-40 85]);

xlabel('Eastings (m)');

ylabel('Northings (m)');

zlabel('Counts');

title('Gridded Dataset');

caxis([2050 2150]);

### Registration function

This function completes an iterative registration process, looping through all frames within the dataset. It requires the frame data and initial *x* and *y* positions, as described previously. It begins by finding the *y* size (# rows) and *x* size (# columns), and pre-allocating the final registered positional matrices. It then assigns the user-defined initial *x* and *y* positions to the first frame's registered positon matrices so that the iterative loop can be run.

function [reg_x,reg_y] = resva_register(frame_data,xpos,ypos)

%Find the x and y resolutions of the frame data

res_x = length(frame_data(1,1,:));

res_y = length(frame_data(1,:,1));

%Initialize the registered position data

reg_x = zeros(length(frame_data(:,1,1)),res_y,res_x);

reg_y = zeros(length(frame_data(:,1,1)),res_y,res_x);

%Assign the GPS positions that we know to the first frame

reg_x(1,:,:) = xpos;

reg_y(1,:,:) = ypos;

Subsequently, the registration function creates and defines the image registration optimizer. There are several variables that can be adjusted by the user, should the default values not work (or be sub-optimal) for a given dataset. See MATLAB's help file on *RegularStepGradientDescent* for more information on each of the specific variables.

%Create the optimizer and metric for the registration

[optimizer, metric] = imregconfig('monomodal'); %Monomodal as data is from a single sensor

optimizer.GradientMagnitudeTolerance = 1e-4; %Default is 1e-4

optimizer.MinimumStepLength = 1e-5; %Default is 1e-5

optimizer.MaximumStepLength = 0.0625; %Default is 0.0625

optimizer.MaximumIterations = 100; %Default is 100

optimizer.RelaxationFactor = 0.5; %Default is 0.5

With the variables and registration setup, the registration function creates a waitbar to track the progress of the iterative process, and then loops through each frame of the dataset, beginning at frame number two, since the initial frame already has positions given by the user.

%Create a waitbar to keep track of iteration number

wait_tracker = waitbar(0,['Iteration# 0 of ' sprintf('%5.0f',length(frame_data(:,1,1)))]);

%Now loop through all frames of data

for i = 2:length(frame_data(:,1,1))

%Update the waitbar

waitbar(i/length(frame_data(:,1,1)),wait_tracker,['Iteration# ' sprintf('%5.0f',i) ' of ' sprintf('%5.0f',length(frame_data(:,1,1)))]);

The fixed frame is set as the current iteration minus one, the moving frame set as the current iteration, and then the registration process is applied. This registration process, given by the MATLAB function *imregtform*, can be constrained by the type of transformation that will be applied to the frame data (e.g. translation, rigid, etc.). In this method, only the transformation ‘*translation*’ can be applied. With some modification to the positional change calculation, other transformations could be added if necessary for a specific dataset.

%Create the indices for the fixed and moving frames

fixN = i-1;

movN = i;

%Create the variables holding the fixed and moving frames

fixedU = reshape(frame_data(fixN,:,:),res_y,res_x);

movingU = reshape(frame_data(movN,:,:),res_y,res_x);

%Apply the registration process

transform_structure = imregtform(movingU, fixedU, 'translation', optimizer, metric);

The output of the registration process is a transformation matrix that, once applied to the fixed frame's positional orientation, will result in the net translations in the *x* and *y* directions.

%Get the fixed frame's position

xfix = reshape(reg_x(fixN,:,:),res_y,res_x);

yfix = reshape(reg_y(fixN,:,:),res_y,res_x);

%Find the average position change across pixels in the x and y directions

xcellx = mean(diff(xfix(1,:)));

xcelly = mean(diff(xfix(:,1)));

ycellx = mean(diff(yfix(1,:)));

ycelly = mean(diff(yfix(:,1)));

%Get the translation amounts from the transform struct

xpixels = transform_structure.T(3,1);

ypixels = transform_structure.T(3,2);

%Calculate the total change in x and y positions

netx = (xpixels * xcellx) + (ypixels * xcelly);

nety = (xpixels * ycellx) + (ypixels * ycelly);

These translations are then applied to the fixed frame's positional data, thus giving the moving frame's new position. Then, this information is assigned to the registered positional matrices, and the loop begins again with the next frame until all frames are complete.

%Apply the translations to the original data, giving the registered frame position

reg_x(i,:,:) = xfix+netx;

reg_y(i,:,:) = yfix+nety;

end

close(wait_tracker); %Close the waitbar before exiting the function

With the registration process complete, the registered *x* and *y* positions for all frames are returned from the function, which will be used for the gridding function.

### Gridding function

Now that registered positions are known for every pixel of every frame, the gridding process combines all frames into a single image. This function requires the frame data, registered *x* and *y* positions, the user-defined cell size, and the user's chosen grid method. The process begins by finding the *y* size (# rows) and *x* size (# columns) of the frame data, followed by determining the total extent of the entire dataset.

function [grid_data,grid_x,grid_y] = resva_grid(frame_data,reg_x,reg_y,cell,gridmethod)

%Find the x and y resolutions of the frame data

res_x = length(frame_data(1,1,:));

res_y = length(frame_data(1,:,1));

%Find the physical extent of the total dataset

minX = ceil(min(min(min(reg_x(reg_x>0))))); %Ensure we ignore any nonsensical negative values that may exist outside the dataset

minY = ceil(min(min(min(reg_y(reg_y>0))))); %Ensure we ignore any nonsensical negative values that may exist outside the dataset

maxX = ceil(max(max(max(reg_x))));

maxY = ceil(max(max(max(reg_y))));

With the dataset's extent found, the total number of cells within the final image is calculated for the *x* and *y* directions. This information is then used to create *x* and *y* matrices of the positions of the image's grid. Temporary matrices that will hold the value and flagging information are pre-allocated, and if the ‘*centerframe*’ method is selected, all cells in the flag matrix are set to a large, positive dummy variable.

%Now use the extent and the user-defined cellsize to determine the total number of cells in the x and y directions.

lengthX = ceil((maxX - minX) / cell)+1;

lengthY = ceil((maxY - minY) / cell)+1;

%Create matrices containing the positions

[grid_y, grid_x] = meshgrid(minY:lengthY+minY-1,minX:lengthX+minX-1);

%Create temporary matrices for the values and associated flags

tempV = zeros(lengthX,lengthY);

tempF = zeros(lengthX,lengthY);

switch gridmethod

case 'centerframe'

tempF(:,:) = 999999; %Set all flags to a dummy variable for initialization

end

Before beginning the loop, a waitbar is initialized to track the iteration progress. The function then opens three loops that will go through all frames, and all individual pixels within each of those frames, while keeping track of the *x* and *y* position of each value.

%Create a waitbar to keep track of iteration number

wait_tracker = waitbar(0,['Iteration# 0 of ' sprintf('%5.0f',length(frame_data(:,1,1)))]);

%Now loop through all frames of data.

for i = 1:length(frame_data(:,1,1))

%Update the waitbar

waitbar(i/length(frame_data(:,1,1)),wait_tracker,['Iteration# ' sprintf('%5.0f',i) ' of ' sprintf('%5.0f',length(frame_data(:,1,1)))]);

%Loop through all pixels of each frame

for j = 1:res_y

for k = 1:res_x

To calculate where in the final image the current pixel will fall, the extent of the dataset and the cell size of the image are used. These *xPos* and *yPos* variables refer to the index within the final image matrices, making it simple to track where data have already been placed within the image.

%First calculate where this pixel falls in the total dataset meshgrid

xPos = floor(abs(reg_x(i,j,k) - minX) / cell) + 1;

yPos = floor(abs(reg_y(i,j,k) - minY) / cell) + 1;

With the indexing information known, the next step is dependent on which grid method was chosen by the user. If the ‘*average’* method is chosen, the pixel's value is added to the cell's total value, and the flag for that cell is increased by one. If the ‘*centerframe*’ is chosen, then the radial distance between the pixel and the frame's center must be calculated. If this radial distance is smaller than any other pixel found previously (as tracked by the flag variable for the cell), or if it is the first pixel found in this cell (as tracked by the dummy variable), then the value of the cell is replaced with the new pixel's value, and the flag is set to the radial distance.

%Now we complete the calculation depending on what gridding

%method was chosen by the user.

switch gridmethod

case 'average'

vals = frame_data(i,j,k);

tempV(xPos,yPos) = tempV(xPos,yPos) + vals;

tempF(xPos,yPos) = tempF(xPos,yPos) + 1;

case 'centerframe'

%We only want to grab the frame whose center is closest to the cell.

xdis = grid_x(xPos,yPos) - reg_x(i,res_y/2,res_x/2);

ydis = grid_y(xPos,yPos) - reg_y(i,res_y/2,res_x/2);

rdis = sqrt((xdis^2) + (ydis^2));

%if the distance is smaller than any currently saved

if rdis < tempF(xPos,yPos)

tempF(xPos,yPos) = rdis;

vals = frame_data(i,j,k);

tempV(xPos,yPos) = vals;

end

otherwise

error('Invalid gridding method chosen!');

end

end

end

end

close(wait_tracker); %Close the waitbar

**Fig. 3:**
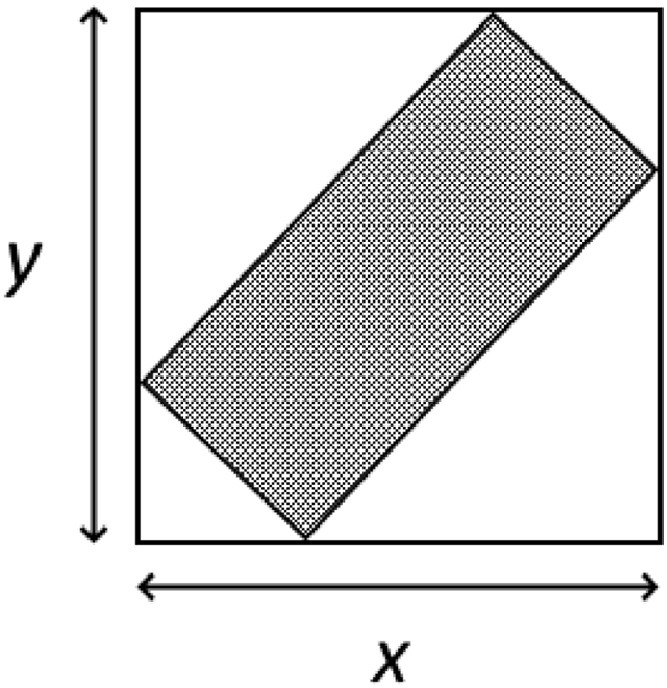
An example of an idealized final gridded dataset. The matrices are of a size (*x,y*), and all cells outside of the highlighted rectangle are set to NaN (the white area).

Once all pixels have been analyzed, a final calculation is performed using the flagging information and based on the gridding method. In the case of the ‘*average*’ method, the final grid data is found by dividing the summed values by the number of values as tracked by the flags. A side-effect of this process is that any cells that do not contain any data (but are still within the total extent of the data) will become Not a Number (NaN) due to the division by 0 in the flag variable (Fig. 3). This is desired as it indicates the bounds of the real data, and is easier for plotting. In the case of the ‘*centerframe*’ method, nothing needs to be done to the completed cells, however the cells outside of the data need to be set to NaNs, which is done by finding all flags that are still the dummy variable.

%Now return the grid_data variable

switch gridmethod

case 'average'

%Take the average of the combined cell data. Note that this will

%inherently set all cells outside of the dataset to NaN (i.e.

%divided by 0).

grid_data = tempV./tempF;

case 'centerframe'

%Manually set all cells outside of the dataset to NaN

tempV(tempF == 999999) = NaN;

grid_data = tempV;

otherwise

error('Invalid gridding method chosen!');

end

With this complete, the gridding function returns the final gridded data, along with the *x* and *y* position matrices for the grid. These three matrices represent the completed process.

### Case study: airborne infrared dataset

**Fig. 4:**
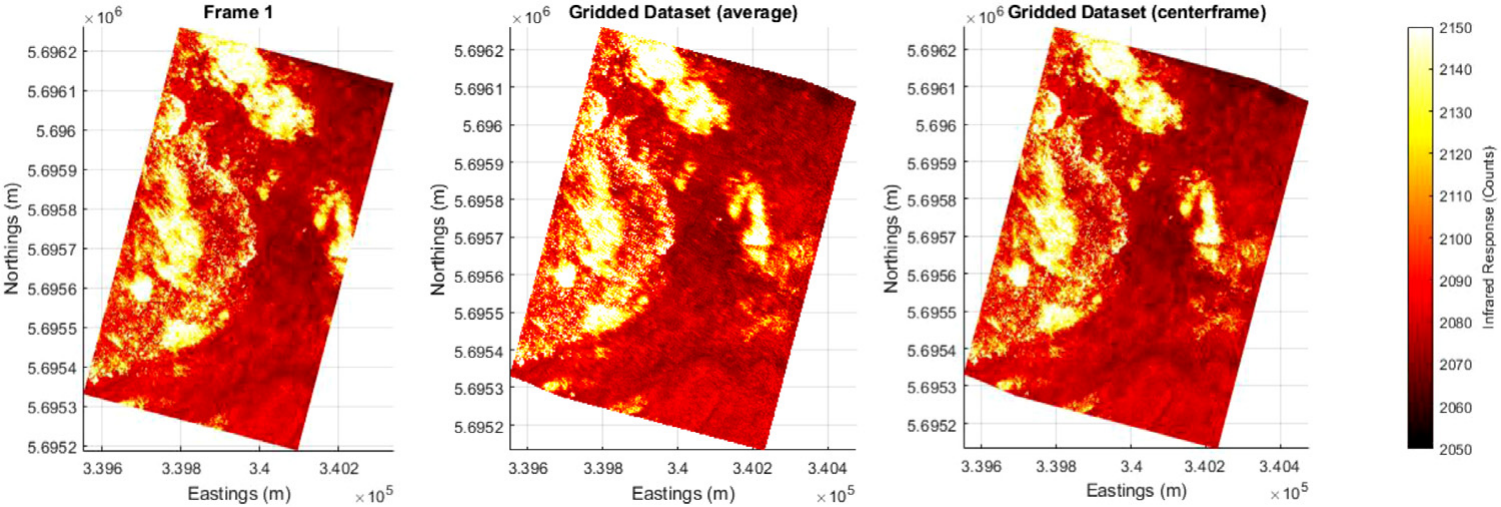
The 13 frame example dataset as processed using the toolbox described here. Note that all three images are plotted with the same colour range, as seen on the far right side. (Left) The first frame with associated GPS information. (Middle) The final result when using the ‘*average*’ method for gridding. (Right) The final result when using the ‘*centerframe*’ method for gridding.

The described method was originally developed for a specific airborne infrared dataset that did not contain proper GNSS and IMU data due to hardware issues [7]. These data were collected by the National Research Council Canada's Twin Otter aircraft near Pickle Lake, ON, Canada, between August 2 and August 3, 2017. A small section of 13 frames are supplied as example data, and the combined solution can be seen in Fig. 4. The dataset takes approximately 43 s to process on a standard desktop with 32 GBs of RAM and an Intel® Xeon® E5-2660 Processor. This method allowed the fire scientists to complete wildfire-specific analysis on the resulting combined imagery.

## Declaration of Competing Interest

The authors declare that they have no known competing financial interests or personal relationships that could have appeared to influence the work reported in this paper.

## Appendix Supplementary materials

Image, application 1

## References

[1] Liu Q., Liu W., Zou L., Wang J., Liu Y. (2012). A new approach to fast mosaic UAV images. Int. Arch. Photogramm. Remote Sens. Spatial Inf. Sci..

[2] Arroyo-Mora J.P., Kalacska M., Inamdar D., Soffer R., Lucanus O., Gorman J., Naprstek T., Schaaf E.S., Ifimov G., Elmer K., Leblanc G. (2019). Implementation of a UAV–hyperspectral pushbroom imager for ecological monitoring. Drones.

[3] Kalacska M., Arroyo-Mora J., de Gea J., Snirer E., Herzog C., Moore T. (2013). Videographic analysis of eriophorum vaginatum spatial coverage in an ombotrophic bog. Remote Sens..

[4] R. Müller, M. Lehner, R. Müller, P. Reinartz, M. Schroeder, B. Vollmer, A program for direct georeferencing of airborne and spaceborne line scanner images, 2002.

[5] Du S., Sun W., Gao Y. (2016). MEMS IMU error mitigation using rotation modulation technique. Sensors.

[6] Stow D.A., Riggan P.J., Storey E.J., Coulter L.L. (2014). Measuring fire spread rates from repeat pass airborne thermal infrared imagery. Remote Sens. Lett..

[7] Ifimov G., Naprstek T., Johnston J.M., Arroyo-Mora J.P., Leblanc G., Lee M.D. (2021). Geocorrection of airborne mid-wave infrared imagery for mapping wildfires without GPS or IMU. Sensors.

